# Safety of COVID-19 Vaccines in Patients With Multiple Sclerosis: A Cross-Sectional Study From a Tertiary Rehabilitation Center in Saudi Arabia

**DOI:** 10.7759/cureus.75889

**Published:** 2024-12-17

**Authors:** Sherif Mohamed, Maaz A Elkarim, Shuja'a A Al-Jaberi

**Affiliations:** 1 Chest Diseases and Tuberculosis, Faculty of Medicine, Assiut University, Assiut, EGY; 2 Internal Medicine, Sultan Bin Abdulaziz Humanitarian City, Riyadh, SAU; 3 Internal Medicine, Bunbury Regional Hospital, Bunbury, AUS

**Keywords:** kingdom of saudi arabia (ksa), observational cross-sectional study, risk factors for multiple sclerosis, safety and health, sars-cov-2 (severe acute respiratory syndrome coronavirus -2), sars-cov-2 vaccines

## Abstract

Background

The safety and adverse effects (AEs) associated with approved COVID-19 vaccines in individuals with multiple sclerosis (MS) require further examination, particularly as there is limited information available for MS patients in Saudi Arabia. This study sought to investigate the reported AEs of COVID-19 vaccines among MS patients admitted to a major rehabilitation center in Saudi Arabia.

Methods

A cross-sectional analysis was conducted from January 2023 to March 2024 at Sultan Bin Abdulaziz Humanitarian City (SBAHC) in Riyadh. All MS patients registered in the electronic medical records were invited to participate in face-to-face or phone interviews. A total of 108 MS patients were surveyed, and data were collected on demographic information, MS-related history (including disease duration, presenting symptoms, and relapses), COVID-19 vaccination status (type, number of doses, and reported AEs), and exposure to disease-modifying therapies.

Results

Among the subjects, there were 58 males (53.7%) and 50 females (46.3%), with a mean age of 38.8 ± 9.7 years, ranging from 17 to 66 years. The relapsing-remitting type of MS was the most prevalent (n=53, 49%). The BNT162b2 mRNA COVID-19 vaccine from Pfizer-BioNTech was the most administered vaccine (n=58, 53.7%). Adverse events were reported by 56 (52%) participants, with the majority being mild (n=79, 73.3%). The ChAdOx1 nCoV-19 vaccine from Oxford-AstraZeneca showed a significantly higher incidence of AEs (n=76, 70%) compared to other vaccines (p < 0.001). Binary logistic regression indicated that the Oxford-AstraZeneca vaccine type was the most significant independent factor associated with COVID-19 vaccination AEs (odds risk 5.337, 95% confidence interval 0.022-18.980, p<0.001).

Conclusions

The safety profile of COVID-19 vaccines in this study is comparable to that of the general population, showing similar rates of mild and self-limiting AEs, with no serious or life-threatening reactions reported. The Oxford-AstraZeneca vaccine was notably linked to a higher incidence of AEs. These findings affirm the safety of COVID-19 immunization in MS patients and reinforce both local and international guidelines to promote vaccination during the COVID-19 pandemic.

## Introduction

Multiple sclerosis (MS) is a demyelinating neurodegenerative disorder of unknown origin, presenting a variety of physical, cognitive, and sometimes psychiatric symptoms [1]. In 2020, the estimated prevalence of MS in Saudi Arabia was approximately 40.40 per 100,000 individuals [2]. The World Health Organization (WHO) Strategic Advisory Group of Experts on Immunization (SAGE) has reaffirmed its prior recommendations regarding COVID-19 vaccination and the necessity of revaccination for high-risk groups [3]. Although many COVID-19 vaccines are deemed safe, some have been associated with significant adverse effects (AEs), particularly thrombotic events and anaphylaxis [4].

The National Multiple Sclerosis Society and other organizations advocate for COVID-19 vaccination in all MS patients, despite prevalent concerns and misconceptions contributing to vaccine hesitancy in this population [5]. Previous research highlighted the potential for increased reactogenicity in MS patients post-vaccination, particularly among younger individuals and those on disease-modifying therapies (DMTs) [6]. While several studies indicate that vaccine responses in MS patients are comparable to those in healthy individuals, the variability in immune response and a lack of extensive clinical trials necessitate a deeper understanding of the safety and efficacy of COVID-19 vaccines in this patient population [2,4,6-9].

Sultan Bin Abdulaziz Humanitarian City (SBAHC) in Riyadh serves as a major rehabilitation facility for patients with various disabilities and neurological disorders [10]. Limited data exists regarding the safety profile of COVID-19 vaccines for MS patients in Saudi Arabia [2,4], prompting the present study to investigate AEs reported by MS patients admitted to this tertiary rehabilitation center.

## Materials and methods

Study design and setting

SBAHC is a prominent rehabilitation hospital that treats patients with various disabilities and neurological disorders, including adults and children. This cross-sectional analysis was conducted at SBAHC between January 2023 and January 2024.

Study population

All MS patients admitted for rehabilitation or receiving outpatient follow-up registered in the facility's electronic medical records were eligible for enrollment (n=124). Participants were approached for face-to-face or phone interviews to complete a questionnaire concerning their COVID-19 vaccination status, demographic details, clinical history, and any AEs experienced post-vaccination. AEs included both local and systemic reactions following each vaccine dose. The AEs were graded according to the FDA’s Toxicity Grading scale as follows: grade 1 (mild), grade 2 (moderate), grade 3 (severe), and grade 4 (serious or life-threatening). Patients with cognitive disabilities (n=6) or those unwilling to participate (n=10) were excluded.

Ethical considerations

All patients gave their informed consent, and the study was approved by the SBAHC Institutional Review Board (Number 65-2022-IRB).

Statistical analysis

Data analysis was performed using SPSS Statistics Software version 27.0 (IBM Corp., Armonk, NY, USA). Chi-square tests were used to compare characteristics of study subjects and the incidence of reactions or side effects following COVID-19 vaccination. Binary logistic regression analysis assessed the correlation between sociodemographic and clinical variables with vaccine side effects. A p-value of <0.05 was considered statistically significant.

## Results

Demographic and clinical data

The study included 108 participants, comprising 58 males (53.7%) and 50 females (46.3%), with a mean age of 38.8 ± 9.7 years (range 17-66 years). The relapsing-remitting subtype was most common (n=53, 49%), followed by primary progressive (n=27, 25%) and secondary progressive (n=22, 20.4%). Fourteen (13%) participants had comorbid medical conditions, while 60 (55.6%) out of 108 were undergoing treatment. Comorbid medical conditions were hypertension, diabetes mellitus, and coronary heart disease in six (5.5%), four (3.7%), and three (2.7%) patients, respectively (Table 1 shows these demographics).

**Table 1:** Demographic and clinical characteristics of the enrolled subjects (n=108) MS, multiple sclerosis

Item	No (%)
Age (years)	
Median, range	37.5, 17-66
Mean ± SD	38.8 ± 9.7
Gender	
Male	58 (53.7)
Female	50 (46.3)
Marital status	
Single	28 (26)
Married	68 (63)
Divorced	12 (11)
Type of MS	
Primary progressive	27 (25.0)
Relapsing-remitting	53 (49.0)
Secondary progressive	22 (20.4)
Clinically isolated syndrome	3 (2.8)
Radiologically isolated syndrome	1 (0.9)
Unknown	2 (1.9)
Duration of MS (years)	
Mean ± SD	13.7 ± 6.7
Range	2-29
Past history of MS relapse	
No	73 (67.5)
Yes	15 (14.0)
Unknown	20 (18.5)
Medical comorbidities	
No	94 (87.0)
Yes	14 (13.0)
COVID-19 vaccination	
No	8 (7.4)
Yes	100 (92.6)
Current MS treatment	
Yes	60 (55.6)
No/unknown	48 (44.4)
Previous adverse effects of injections other than COVID-19 vaccine	
Yes	12 (11.0)
No	73 (67.5)
Unknown	23 (21.5)

Motor symptoms, such as difficulties with walking, spasticity, and hand function, were reported by 79 (73%) of 108 patients, with sensory symptoms (n=68, 63%) and fatigue (n=66, 61.1%) also prevalent. Many patients reported multiple symptoms.

Safety profile and adverse events of the given COVID-19 vaccines

**Figure 1:**
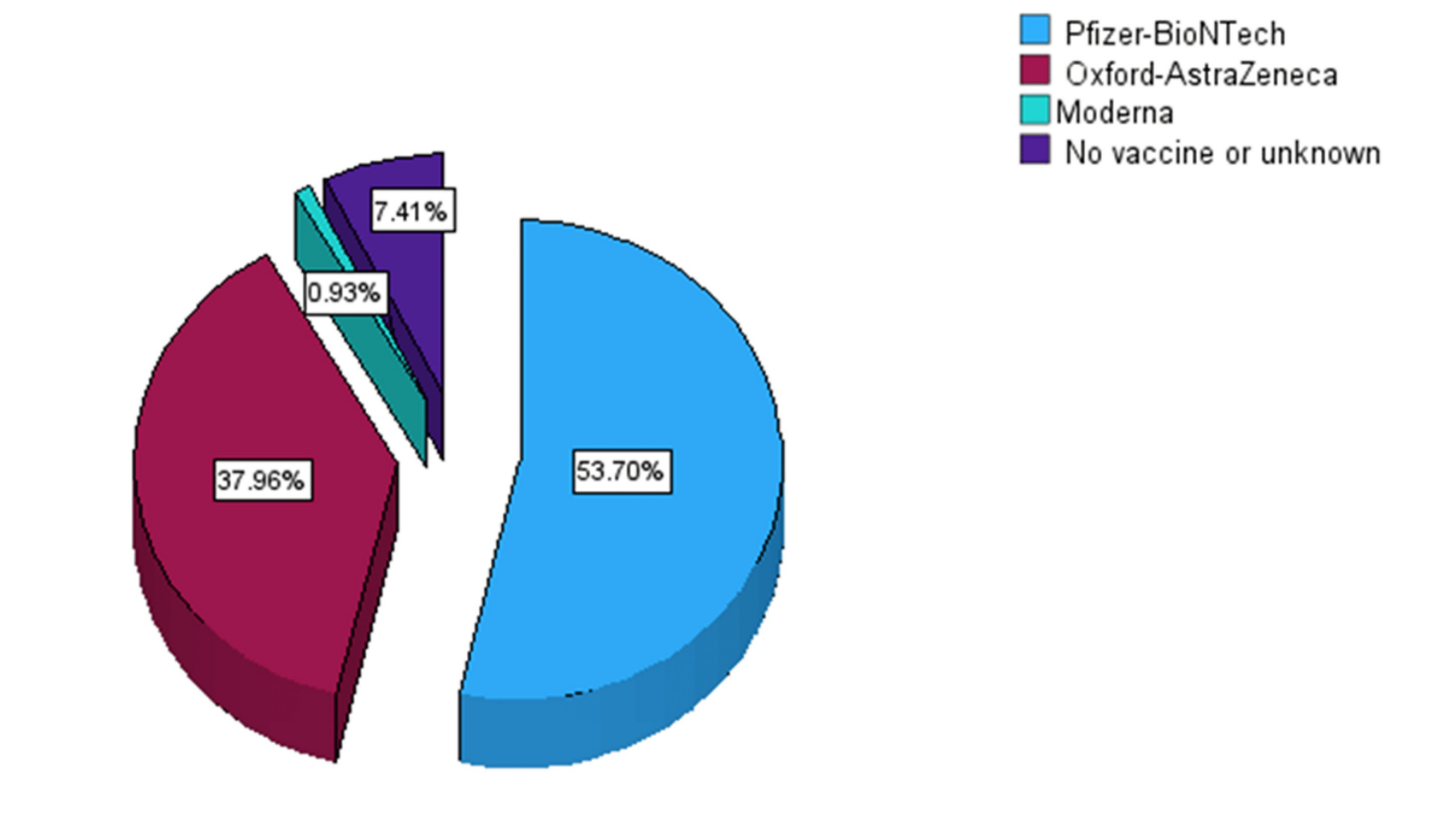
Types of COVID-19 vaccines given

A total of 100 (92.6%) patients received one or more COVID-19 vaccine doses. The BNT162b2 mRNA vaccine from Pfizer-BioNTech was the most frequently administered (n=58, 53.7%), followed by the ChAdOx1 nCoV-19 vaccine from Oxford-AstraZeneca (n=41, 38.0%) and Moderna (n=1, 0.9%) (Figure 1).

Five (4.6%) patients received a single vaccine dose, while 27 (25.0%) and 56 (52.0%) received two and three doses, respectively. Adverse events were reported by 56 (52%) participants within two days (n=88, 81%) and up to one week (n= 8, 7%), with common reactions including headache (n=41, 38%), pain or tenderness (n=82, 76%), fatigue (n=72, 66.7%), fever (n=63, 58%), and muscle aches (n=68, 63%). Most reported AEs were mild (n=79, 73.3%), while moderate and severe AEs were noted in 27 (24.7%) and 3 (2.0%) subjects, respectively. Two (1.8%) patients experienced MS relapses within two days post-vaccination. They was one 58-year-old female who had MS relapse after receiving the second dose of Oxford-AstraZeneca vaccine. She was on teriflunomide. She had MS for 18 years and experienced MS one time before. The second patient was a 32-year-old male who had MS relapse after receiving the third dose of the Pfizer-BioNTech vaccine. He was on rituximab. He had MS for eight years and had no history of relapse before. Both patients needed to visit their neurologist for this relapse. Six (5.0%) patients reported worsening of pre-existing MS symptoms, which were transient and related to mild fever (Table 2).

**Table 2:** Adverse effects or reactions of COVID-19 vaccines (n=302)* *This represents the total number of reactions or adverse effects, not the patients MS, multiple sclerosis

Variable	No (%)
Immediate allergic reaction	2 (0.6)
Pain, soreness, or tenderness at the injection site	82 (27.0)
Fever	63 (20.8)
Dizziness	33 (11.0)
Chills	10 (3.0)
Fatigue	72 (23.8)
Headache	41 (13.5)
Muscle aches	68 (22.5)
Malaise	75 (24.8)
Joint pain	22 (7.0)
Shortness of breath	9 (3.0)
Redness (injection site)	33 (11.0)
Swelling (injection site)	16 (5.0)
Itching (injection site)	12 (4.0)
Menstrual cycle changes	2 (0.6)
Nausea	4 (1.0)
MS relapse	2 (0.0)
Worsening of pre-existing MS symptoms	16 (5.0)
None	46 (15.0)

Association between the vaccines’ adverse events and the clinical/demographic characteristics

The study evaluated the correlation between patient characteristics (age, gender, MS type and duration, comorbidities, and previous relapses), vaccine-related factors (vaccine type and doses), and AEs. The Oxford-AstraZeneca vaccine showed a significant association with a higher incidence of AEs (39/56, 70.0%) compared to other vaccines (p < 0.001). Other factors such as age, gender, MS type, number of doses, and treatment for MS did not display significant associations with vaccine AEs (Table 3).

**Table 3:** Association between the COVID-19 vaccine adverse events and clinical/demographic variables *A p-value of <0.05 is considered statistically significant MS, multiple sclerosis

Variable	Adverse effects of COVID-19 vaccines		p-Value*
	Yes (no %)	No (no %)	
	56 (52%)	52 (48%)	
Age groups			
<30 years	10 (18)	7 (13)	0.603
≥30 years	46 (82)	45 (87)	
Gender			
Males	29 (52)	29 (56)	0.703
Females	27 (48)	23 (44)	
Type of MS			
Primary progressive	15 (27)	12 (23)	0.494
Relapsing-remitting	27 (48)	26 (50)	
Secondary progressive	10 (18)	12 (23)	
Clinically isolated syndrome	3 (5)	0 (0)	
Radiologically isolated syndrome	0 (0)	1 (2.0)	
Unknown	1 (2)	1 (2.0)	
MS duration			
<15 years	30 (53.5)	29 (56)	0.849
≥15 years	26 (46.5)	23 (44)	
Doses of COVID-19 vaccines			
One	4 (7)	1 (2)	0.595
Two	13 (23)	14 (27)	
Three	28 (50)	28 (54)	
No/unknown	11 (20)	9 (17)	
Type of the vaccine			
Pfizer-BioNTech	17 (30)	41 (79)	0.001
Oxford-AstraZeneca	39 (70)	2 (4)	
Moderna	0 (0)	1 (2)	
No/unknown	0 (0)	8 (15)	
Comorbidities			
Yes	10 (18)	4 (8)	0.155
No	46 (82)	48 (92)	
Previous MS relapse			
Yes	10 (18)	5 (10)	0.264
No	34 (61)	39 (75)	
Unknown	12 (21)	8 (15)	
MS treatment			
Ocrelizumab	9 (16)	15 (29)	0.165
Dimethyl fumarate	5 (9)	0 (0)	
Terifluonomide	3 (5)	1 (2)	
Rituximab	3 (5)	3 (6)	
Beta-interferons	4 (7)	7 (13)	
Fingolimod	3 (5)	1 (2)	
Almetuzumab	0 (0)	1 (2)	
Glatiramer acetate	1 (2.5)	0 (0)	
Natalizumab	1 (2.5)	3 (6)	
No/unknown	27 (48)	21 (40)	

Binary logistic regression analysis identified the Oxford-AstraZeneca vaccine as the sole significant factor linked to COVID-19 vaccination AEs (odds risk 5.337, p< 0.001) (Table 4).

**Table 4:** Association between the COVID-19 vaccine adverse events and clinico-demographic variables (binary logistic regression analysis) *A p-value of <0.05 is considered statistically significant OR, odds ratio; MS, multiple sclerosis

Variable	OR (95% confidence interval)	p-Value*
Age group (≥30 years)	0.236 (0.030–1.862)	0.171
Gender (female)	0.764 (0.330–1.761)	0.562
Type of the vaccine (AstraZeneca)	5.337 (0.022–18.980)	<0.001
Number of doses of the vaccine	1.272 (0.534–3.029)	0.587
Comorbidities (yes)	0.864 (0.046–1.687)	0.164
Type of MS	0.779 (0.435–1.395)	0.400
Duration of MS	0.867 (0.647–1.161)	0.337
Previous MS relapse (yes)	0.554 (0.282–1.092)	0.088

## Discussion

This study aimed to evaluate the safety profile and AEs of COVID-19 vaccines in MS patients admitted or treated at a large rehabilitation center in Saudi Arabia. The clinical and demographic characteristics of the participants were consistent with typical profiles of MS patients globally, with the relapsing-remitting subtype being the most common. The mean age of participants was 38.8 ± 9.7 years, and the mean disease duration was 13.7 ± 6.7 years. More than half of the patients (55.6%) were actively receiving immunomodulatory therapy with various DMTs.

Only 14 (13.0%) of patients had medical comorbidities, which could be explained by the relatively young age of enrolled subjects. Moreover, the symptoms of enrolled subjects align with the previously reported and known figures for autonomic, visual, motor, and sensory problems [1]. Also, 66 (61.0%) of our cohorts reported that fatigue was a predominant symptom at presentation, which agrees with previous studies [11].

Of our enrolled cohorts, 52% experienced one or more adverse events after the administration of the COVID-19 vaccines. This is typically similar to those figures reported previously [12,13]. In a recently published meta-analysis of 19 observational studies comprising 14,755 MS patients who received 23,088 COVID-19 vaccines, Stefanou et al. [13] observed that adverse events were reported in 52.8% of MS patients. However, our reported COVID-19 vaccine-related AEs are less than those reported by studies from Saudi Arabia [2] and Kuwait [9]. This might be attributable to the differences in enrolled subjects' number, clinical and demographic characteristics, and the effects of the currently used DMTs.

Importantly, our reported rates of vaccine-related AEs are similar to those rates reported from the normal population [14,15], real-life studies on MS patients [16], and MS patients who used other vaccinations [17]. Therefore, these findings suggest that patients with MS did not have a higher risk for vaccine-induced adverse events. Local reactions such as redness, pain, and swelling at the injection site were the most common encountered local adverse events [14,15].

In our MS cohort, fatigue was reported in 72 (76.0%), headache in 41 (38.0%), and pain, soreness, or tenderness in 82 (76.0%). Our results agree with those of Achiron et al. [18], who reported fatigue in 20% of patients and headache in 9% of patients with MS. Notably, most reported adverse events in our cohorts were mild.

Six (5%) of our participants reported worsening of pre-existing MS symptoms following the vaccination. This concurred with the results by Alroughani et al. [9]. However, our results are lower than those of Lotan et al. [16], who reported a 15% worsening of MS symptoms. The low rate of worsening pre-existing MS symptoms, in addition to its overall mild severity, supports previous studies on other non-live-attenuated vaccines that were not related to an increased risk of worsening MS symptoms [19].

We reported MS relapse in two (1.8%) cases after receiving the second dose of Oxford-AstraZeneca and the third dose of Pfizer-BioNTech. Alroughani et al. [9] reported MS relapse in five (1.3%) cases shortly after the first dose of the vaccine, three cases after Pfizer-BioNTech, and two cases after Oxford-AstraZeneca vaccines. Similarly, Maniscalco et al. [20] reported a female patient presented with weakness in her left side of the body and paresthesia two days after receiving the mRNA COVID-19 vaccine.

The association between the patient's clinical characteristics, vaccine-related factors, and the AEs of COVID-19 vaccination revealed interesting results.

The Oxford-AstraZeneca vaccine was significantly associated with a higher incidence of adverse events (70%) than the other vaccines. Other factors, such as age, gender, type of MS, number of COVID-19 vaccine doses, and treatment for MS, were not significantly associated with AEs of COVID-19 vaccines. The type of vaccine (manufactured by Oxford-AstraZeneca) was the only significant factor associated with AEs of COVID-19 vaccination (odds risk 5.337, p< 0.001).

This was consistent with the findings of the recent Saudi study by Aladdin et al. [2]. Similarly, Briggs et al. [6], in a large study involving 718 patients, found a higher prevalence in patients receiving the Oxford-AstraZeneca vaccine compared to those who received Pfizer-BioNTech and mRNA-1273 SARS-CoV-2 vaccines.

Notably, our results were similar to those of Aladdin et al. [2] and Alroughani et al. [9], denoting no significant association between the adverse events of the COVID-19 vaccines and the use of DMT. Previous studies observed that COVID-19-vaccinated MS patients can develop protective humoral responses [21].

The current study's results showed that COVID-19 vaccines were tolerated in our cohorts with MS, and the side effects were comparable to those reported in other countries [9,12,13,16,18,22]. The results of the current study could have important clinical implications.

Given the tolerability and acceptable side effects of COVID-19 vaccines, together with the negative impacts of COVID-19 and its long-term sequelae on patients with MS, patients with MS are highly encouraged to receive COVID-19 vaccines. Weighted against the risks of SARS-CoV-2-related complications and MS exacerbations, these safety data provide compelling pro-vaccination arguments for MS patients.

Our findings provide a piece of additional safety information related to COVID-19 vaccination in patients with this neurological disorder and support the given recommendations to encourage and not delay vaccination for the COVID-19 pandemic. Moreover, our study highlights the supportive analysis of the benefits/risk assessment against possible minor risks of COVID-19 vaccines in those patients actively using DMTs. More prospective multicenter studies should validate these findings to assess the longstanding sequelae of COVID-19 vaccines in MS patients.

Limitations

The study's limitations include its single-center design, which may restrict the generalizability of the results. Additionally, the analysis focused solely on short-term adverse events, with plans for future evaluations to assess long-term effects.

## Conclusions

The findings indicate that the safety profile of COVID-19 vaccines in MS patients is comparable to the general population, with similar rates of minor, self-limiting AEs. The Oxford-AstraZeneca vaccine was significantly associated with a higher incidence of adverse events compared to other vaccines and was the primary independent factor linked to these effects. The results support the safety of COVID-19 immunization in MS patients and advocate for timely vaccination against COVID-19. Further multicenter prospective studies are warranted to explore these findings comprehensively.
